# Supplementary material for: When weight is an encumbrance; avoidance of stairs by different demographic groups
Source: PLoS One. 2020 Jan 24;15(1):e0228044. doi: 10.1371/journal.pone.0228044 (PMC6980638; doi:10.1371/journal.pone.0228044)
Supplement: S1 File — (PDF) [file pone.0228044.s001.pdf]

Supplementary file 1: When weight is an encumbrance; avoidance of stairs by different demographic groups.

Previous studies of the effects of demographic grouping on stair avoidance at work.

This summary results from a literature review of previously reported effects of demographics on choice between stairs and elevators at work. As well as weight status, two further demographic features related to weight were included in this summary. Carrying a large bag upstairs would increase the weight carried against gravity and should bias behaviour towards avoidance. In addition, women have, on average, a greater percentage of their weight as body fat than men, i.e., carry more dead weight, yet have lower leg strength that will be used to raise weight against gravity (McCardle et al., 2007). As a result of these overall differences, a woman climbing the stairs would require more of her available physiological resources for climbing relative to a man of the same weight. Both carrying a large bag and being female were consistently associated with greater avoidance of stairs in public access settings and this summary tested whether there was evidence for similar effects at work (Eves, 2014).

To review the available data, searches of the online electronic data bases, MEDLINE; Cumulative Index to Nursing & Allied Health Literature _ CINAHL; and PsycINFO using the keywords combinations; ‘stair climbing’, ‘stair use’, and the terms used to describe stair interventions ‘point-of-choice’ and ‘point-of-decision’, retrieved 93 articles on stair use in the environment, with 45 covering stair use in workplaces. The constraint for inclusion was at least one coded demographic. Twenty one separate studies provided relevant information. Table 1 below summarizes the statistically significant effects of demographics on stair avoidance in each study. The column labeled ‘Multi’ indicates whether the results were from multivariate analyses. Natural variation in the pedestrians using a site means that coding for multiple demographics may not be fully independent. Carrying a large bag may co-vary with sex and multivariate analyses are preferable. Additionally, studies that analyse avoidance of the more costly behavior of stair climbing better address the question than those that combine stair climbing and descent into stair use before analysis.

Supplementary Table 1: Summary of the effects of demographic grouping on avoidance of stairs in the workplace in previous research.

Names (date)	Site	n =	Baseline % elevator	Multi	Stair measure	Overweight vs. Not	Bag vs. No bag	Females vs. Males
Russell, Dzewaltowski & Ryan (1999)	University library (U.S.)	6,216	60.3	yes	climbing	-	exclude	F>M
Kerr, Eves & Carroll (2001a)	Office (U.K.)	12,288	79.3	yes	climbing	-	B>NB	M>F
	Office (U.K.)	2,694	81.0	yes	climbing	-	=	F>M
Coleman & Gonzalez (2001)	University library (U.S.)	15,233	64.2	no	climbing	-	-	F>M
	Office (U.S.)	8,361	66.8	no	climbing	-	-	M>F
Boutelle, Jeffrey, Murray & Schmitz (2001)	Health promotion facility (U.S.)	35,475	-	no	use	-	-	M>F
Eves, Webb & Mutrie (2006)	Office (U.K.)	26,806	70.9	yes	climbing	OW>N	B>NB	F>M
Bungum, Meacham & Truax (2007)	Two uni., five banks, one garage (U.S.)	2,050	83.5	yes	use	-	exclude	=
Kwak, Kremers, van Baak & Brug (2007)	Office & factory combined (Holland)	6,771	-	yes	use	-	exclude	M>F
Slaunwhite, Smith, Fleming, & Fabrigar (2009)	University (Canada)	3,339	17.0	yes	climbing	-	exclude	=
Pillay, Kolbe-Alexander, Achmat, Carstens & Lambert (2009)	Sport medicine institute (South Africa)	4,256	57.0	no	climbing	-	-	=
Grimstvedt, Kerr, Oswalt, Fogt, Vargas -Tonsing, & Yin (2010)	Four university buildings (U.S.)	8,431	-	yes	use	-	exclude	F>M

Andersen (2010)	Four university buildings (U.S.)	2,707	-	yes	use	-	=	F>M
van Nieuw-Amerogen, Kremers, de Vries & Kok (2011)	University (Holland)	21,786	48.2	no	climbing	-	-	F>M
Olander & Eves (2011a)	Four university buildings (U.K.)	4,279	52.1	yes	climbing	-	-	F>M
Howie & Young (2011)	Two university dormitories (U.S.)	6,338	75.1	no	climbing			=
Lewis & Eves (2012)	Four university buildings (U.K.)	14,138	40.6	yes	climbing	-	-	F>M
Chhay (2013)	University building (U.S.)	2,862	47.2	no	climbing	-	exclude	M>F
	Garage (U.S.)	2,472	43.7	no	climbing	-	exclude	=
Nomura, Katayama, Kashiwa, Akezaki & Sato (2014)	Office building (Japan)	16,583	28.4	no	climbing	-	-	=
Eckhardt, Kerr & Taylor (2015)	University nursing building (U.S.)	2,680	88.7	yes	climbing	OW>N[a]	B>NB	=
	Subtotal	205,765				2/2	3/5	9/21

Multi indicates whether the results were from multivariate analyses and **Stair measure** indicates whether the results were specific to stair climbing or combined climbing and descent for analysis (use). For the body of the table, **Uni**. = University, **=** indicates no significant differences between the demographic groups in that column, **-** indicates that the information is unavailable or the demographic grouping was not coded in that study, **>** indicates that one group avoided stairs more than the other, e.g. F > M means females avoided stairs more than men. **F** =

females, **M** = males, **B** = indicates those carrying a large bag/load, **NB** = those without a large bag/load, **exclude** indicates that individuals encumbered with a large bag were not coded in that study, **OW** = overweight pedestrians, **N** = not overweight.

a Eckhardt et al., (2015) coded obese vs. not but significantly greater avoidance was reported in the heavier group.

As can be seen from the table, very few studies have recorded anything other than sex of the pedestrian at work. The two studies with data on the effect of weight status, and three out of the five recording the presence of large bags, reported greater avoidance of stairs in overweight and encumbered pedestrians respectively, a pattern that would be consistent with greater avoidance by these demographic groups in public access settings. Effects for sex on avoidance were mixed. Nine reported that females avoided stairs significantly more than males whereas five reported the opposite disparity and seven no significant difference. There were no obvious co-occurrences of sex differences in avoidance with multivariate analysis or the choice to analyse stair use, rather than climbing alone, that might explain the mixed evidence.

One further point from the table is important. Typically, stair use is more common at work that in public access settings (Eves & Webb, 2006). The table included the percentage choosing the elevator at baseline in each study when reported to illustrate this point. The sample size weighted average of the avoidance, 61.9%, means that expending energy climbing at work (38.1%) was more than five times more common than it was in shopping malls. Any bias against expenditure was reduced at work. The main body of the paper provides a cogent explanation for this difference between settings.

References

1) McCardle WD, Katch FI, Katch VL. *Exercise Physiology, 6th Edition.* Philadelphia, US: Lippincott, Williams & Wilkins. 2007

2) Eves FF. Is there any Proffitt in stair climbing? A headcount of studies testing for demographic differences in choice of stairs. *Psychonom Bull Rev* 2014, 21, 71-79.

3) Eves FF, Webb OJ. Worksite interventions to increase stair climbing; Reasons for caution. *Prev Med* 2006, 43:4-7.

Studies in the table

Russell WD, Dzewaltowski DA, Ryan GJ. The effectiveness of a point-of-decision prompt in deterring sedentary behaviour. *Am. J. Health Prom* 1999, **13**:257-259.

Kerr J, Eves FF, Carroll D. Can posters prompt stair use in a worksite environment? *J Occ Health* 2001a, **43**:205-207.

Boutelle, K.N., Jeffrey, R.W., Murray, D.M., & Schmitz, M.K., 2001. Using signs, artwork, and music to promote stair use in a public building. *Am J Health Prom* **91**:2004-2006.

Coleman KJ, Gonzalez EC. Promoting stair use in a U.S.-Mexico border community. *Am J Pub Health* 2001, **91:**2007-2009.

Eves FF, Webb OJ, Mutrie N. A workplace intervention to promote stair climbing: greater effects in the overweight. *Obes* 2006, **14:**2210-16.

Bungum T, Meacham M, Truax N. The effects of signage and the physical environment on stair usage*. J Phys Act Health* 2007, **4**:237-244.

Kwak L, Kremers SPJ, van Baak MA, Brug J. Prompts promote stair use in blue- and white-collar worksites. *Prev Med 2007*, **45**:177-181.

Pillay JD, Kolbe-Alexander T, Achmat M, Carstens M, Lambert EV. Are point-of-decision prompts in a sports science and medicine centre effective in changing the prevalence of stair usage? A preliminary study. *South African J Sports Med* 2009, **21**:58-64.

Slaunwhite JM, Smith SM, Fleming MT, Fabrigar LR. Using normative messages to increase healthy behaviours. *Int J Workplace Health Manag* 2009, **2**:231-244.

Andersen, L (2010) Effects of environmental prompts on stair usage. (Unpublished thesis) University of Nevada. *UNLV Theses/Dissertations/Professional Papers/Capstones.* Paper 772.

Grimstvedt ME, Kerr J, Oswalt SB, Fogt DL, Vargas-Tonsing TM, Yin Z. Using signage to promote stair use on a university campus in hidden and visible stairwells. *J Phys Act Health* 2010*,* **7**:232-238.

Howie EK, Young DR. Step it up: A multi-component intervention to increase stair use in a university residence building. *Am J Health Prom* 2011, **26**:2-5.

Olander E, Eves FF. Cost and effectiveness of two stair climbing interventions - less is more. *Am J Health Prom* 2011a*,* **25:**231-236.

van Nieuw-Amerongen M E, Kremers SPJ, de Vries NK, Kok G. The use of prompts, increased accessibility, visibility, and building aesthetics of the stairwell to promote stair use in a university. *Envir Behav* 2011, **43**:131-139.

Lewis A, Eves FF. Prompt before the choice is made; Effects of a stair climbing intervention in university buildings. *Brit J Health Psych* 2012, **17**:631-43.

Chhay N. (2013) *Use of stair prompts to encourage physical activity.* (Unpublished thesis). San Jose, USA: San Jose State University

Nomura T, Katayama K, Kashiwa T, Akezaki Y, Sato A. Maintenance of the rate of stair use over a long-term period using a stair climbing campaign *J Occ Health* 2014, 56: 511–518.

Eckhardt MR, Kerr J, Taylor WC. Point-of-decision signs and stair use in a university worksite setting: General versus specific messages. *Am J Health Promot* 2014; 29(5):291-293.
