# Supplementary material for: When weight is an encumbrance; avoidance of stairs by different demographic groups
Source: PLoS One. 2020 Jan 24;15(1):e0228044. doi: 10.1371/journal.pone.0228044 (PMC6980638; doi:10.1371/journal.pone.0228044)
Supplement: S2 File — (PDF) [file pone.0228044.s002.pdf]

Supplementary file 2: When weight is an encumbrance; avoidance of stairs by different demographic groups.

Building characteristics for study 1

Table 1: Building characteristics, sample sizes and % avoidance of stairs by using the elevator for each direction of travel.

Name	Employees	Floors	Elevators	Up n =	Elevator up %	Down n =	Elevator down %
Civic Centre	Local government	4	1	1,047	29.1	872	13.8
Thames House	Commercial	4	1	2,275	39.6	1,746	17.2
Sutton House	Local government	4	2	2,625	41.9	2,057	26.4
Christchurch House	Local government	5	2	2,702	26.6[a]	2,481	18.5
Chamber of Commerce	Commercial	6	2	1,827	59.6	1,483	30.4
Broadgate House	Local government	6	2	1,932	54.6	2,022	25.9
Triplex House	Commercial	10	2	2,199	76.0	1,673	62.8
	Total			14,607	46.8[b]	12,334	28.0

a The stairs in this building were reached some 7m before the elevators and represented the shorter route between the entrance and the upper floors. Distance minimization in buildings is associated with reduced elevator avoidance (Olander & Eves, 2011).

b Sample-size weighted average of avoidance.

Reference

Olander E, Eves FF. Cost and effectiveness of two stair climbing interventions - less is more. *Am J Health Prom* 2011, **25:**231-236.
