# Supplementary material for: When weight is an encumbrance; avoidance of stairs by different demographic groups
Source: PLoS One. 2020 Jan 24;15(1):e0228044. doi: 10.1371/journal.pone.0228044 (PMC6980638; doi:10.1371/journal.pone.0228044)
Supplement: S3 File — (PDF) [file pone.0228044.s003.pdf]

Supplementary File 3: When weight is an encumbrance; avoidance of stairs by different demographic groups.

Outdoor sites where the alternative to stairs was a sloped ramp.

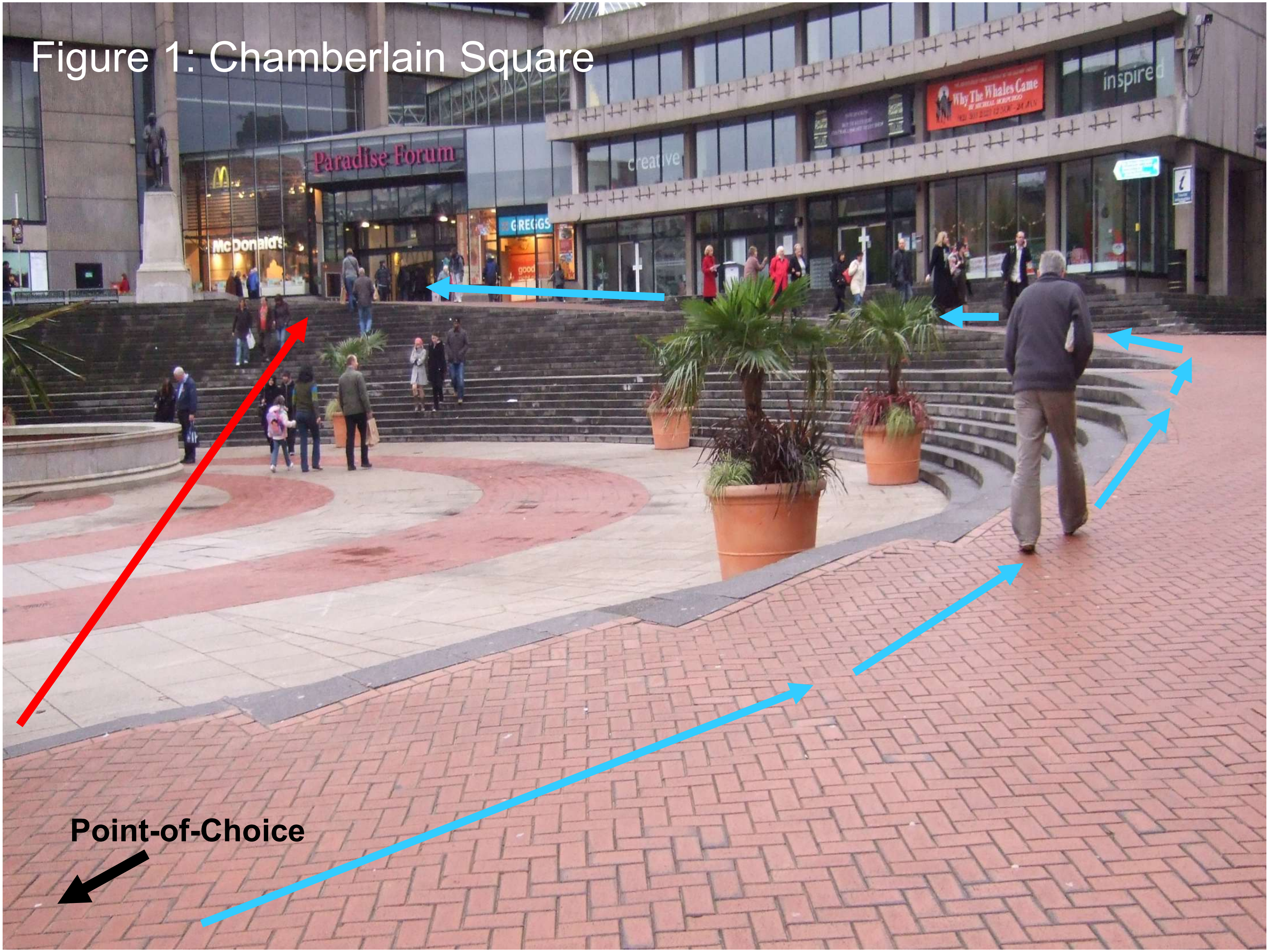

Figure 1: Chamberlain Square

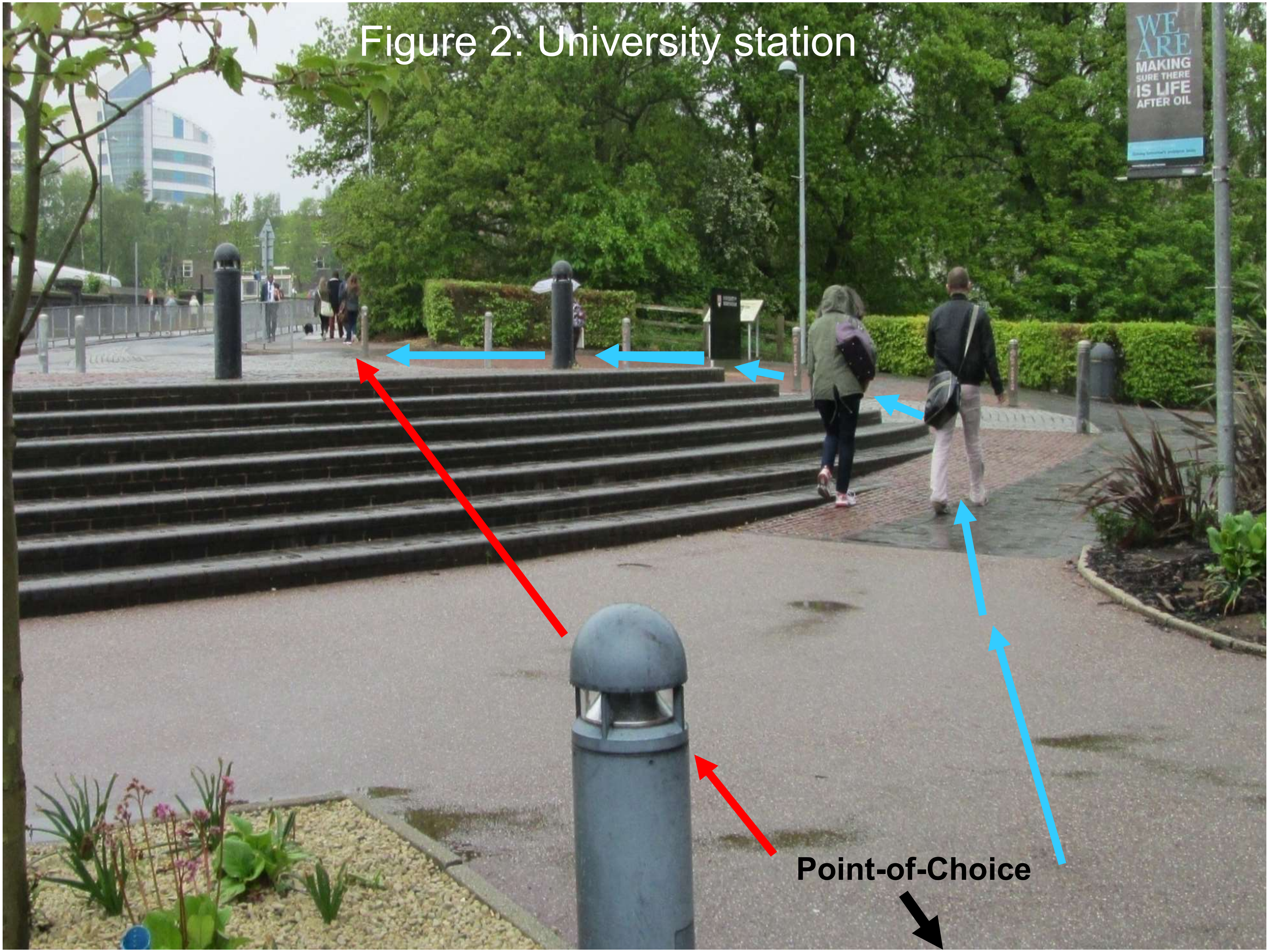

Figure 2: University station
